# Environmental epidemiology of Kawasaki disease: Linking disease etiology, pathogenesis and global distribution

**DOI:** 10.1371/journal.pone.0191087

**Published:** 2018-02-07

**Authors:** Cedric Manlhiot, Brigitte Mueller, Sunita O’Shea, Haris Majeed, Bailey Bernknopf, Michael Labelle, Katherine V. Westcott, Heming Bai, Nita Chahal, Catherine S. Birken, Rae S. M. Yeung, Brian W. McCrindle

**Affiliations:** 1 Labatt Family Heart Centre, Department of Pediatrics, The University of Toronto, Hospital for Sick Children, Toronto, Ontario, Canada; 2 Division of General Pediatrics, Department of Pediatrics, The University of Toronto, Hospital for Sick Children, Toronto, Ontario, Canada; 3 Division of Rheumatology, Department of Pediatrics, The University of Toronto, Hospital for Sick Children, Toronto, Ontario, Canada; Kaohsiung Chang Gung Memorial Hospital, TAIWAN

## Abstract

**Background:**

The pathogenesis of Kawasaki disease (KD) is commonly ascribed to an exaggerated immunologic response to an unidentified environmental or infectious trigger in susceptible children. A comprehensive framework linking epidemiological data and global distribution of KD has not yet been proposed.

**Methods and findings:**

Patients with KD (n = 81) were enrolled within 6 weeks of diagnosis along with control subjects (n = 87). All completed an extensive epidemiological questionnaire. Geographic localization software characterized the subjects’ neighborhood. KD incidence was compared to atmospheric biological particles counts and winds patterns. These data were used to create a comprehensive risk framework for KD, which we tested against published data on the global distribution. Compared to controls, patients with KD were more likely to be of Asian ancestry and were more likely to live in an environment with low exposure to environmental allergens. Higher atmospheric counts of biological particles other than fungus/spores were associated with a temporal reduction in incidence of KD. Finally, westerly winds were associated with increased fungal particles in the atmosphere and increased incidence of KD over the Greater Toronto Area. Our proposed framework was able to explain approximately 80% of the variation in the global distribution of KD. The main limitations of the study are that the majority of data used in this study are limited to the Canadian context and our proposed disease framework is theoretical and circumstantial rather than the result of a single simulation.

**Conclusions:**

Our proposed etiologic framework incorporates the 1) proportion of population that are genetically susceptible; 2) modulation of risk, determined by habitual exposure to environmental allergens, seasonal variations of atmospheric biological particles and contact with infectious diseases; and 3) exposure to the putative trigger. Future modelling of individual risk and global distribution will be strengthened by taking into consideration all of these non-traditional elements.

## Introduction

Kawasaki disease (KD) is a complex, limited, systemic vasculitic syndrome of unknown etiology that mainly occurs in infants and toddlers, and potentially causes severe coronary artery aneurysms [1]. Since its initial identification by Tomisaku Kawasaki in 1970, epidemiological surveys have been periodically conducted in many countries to monitor the worldwide distribution of KD and to attempt to elucidate its cause [2–4]. The high incidence of KD in Asian populations globally is strong evidence of a genetic contribution to disease susceptibility. Significant familial associations have been found in KD with a higher incidence rate being reported in children with family members who have previously had an episode of KD [5–7]. Genome wide linkage studies have identified multiple regions that are associated with an increased risk of KD, and genetic polymorphisms for increased risk of KD, further, increased risk of coronary artery abnormalities resulting from KD have been identified [8–11]. The current consensus is that KD is likely the result of an exaggerated immune response to an environmental or infectious trigger occurring in genetically susceptible children.

The prominent seasonal and temporal/spatial distribution of KD cases has led to many theories suggesting an infectious trigger to the disease [12]. Despite an important body of research, no substantial, reproducible study has been to able to identify a specific infectious trigger(s), even though an important proportion of KD cases are associated with an infectious co-diagnosis at presentation [13]. Previous studies performed in the 1980’s and 1990’s have uncovered potential associations between environmental factors and risk of KD [14–16] but there is a critical lack of contemporary, high quality, environmental epidemiology studies on KD. More recently, studying tropospheric wind patterns and using a flexible particle dispersion model (Flexpart) for backtracking winds over Japan, Rodo *et al*. identified northeastern China as a potential source region for a trigger, and suggested that, due to the short incubation time, a toxic or microbial antigen agent is most likely [17]. Such an agent might possibly be carried over the Pacific Ocean, and a zonal wind pattern was found to be associated with increased KD [18]. The study of the environmental epidemiology of KD might discover additional clues, either to identify the trigger for KD or to elucidate modulatory mechanisms that may increase or decrease the risk of developing KD when encountering said trigger. In this study, we sought to determine the environmental factors associated with both individual risk of KD, local epidemiological patterns, and to integrate these into a comprehensive model to explain the global distribution.

## Materials and methods

### Environmental epidemiology study

The first component of this study comprised a case-control observational study enrolling patients with a new diagnosis of KD within 6 weeks of hospital discharge after their acute admission. Patients were approached either during a follow-up clinic visit or by mail. Controls subjects were recruited who had an unremarkable recent medical history and who were not taking regular medication. These subjects were recruited using a variety of means (in person; e-mail invitation), but the majority of controls were recruited through the TARGet Kids! Collaborative [19], which enrolls and follows healthy children from birth to 10 year old at the time of their regularly scheduled well-child visits. Enrollment of control subjects was stratified by age and gender to reflect the age and gender distribution of patients with KD.

All parents were asked to complete a detailed paper-based questionnaire regarding all aspects of their child’s medical history, immediate living environment and important events, clinical signs and symptoms in the weeks prior to the onset of KD symptoms. Details of the questionnaire are provided in Table 1. Many of the questions regarding events or conditions were dependent on close association with the time since the onset of KD symptoms. Therefore, we limited enrollment to 6 weeks after discharge from the acute admission in order to reduce important recall bias. For control subjects, we had to simulate the timing of events to cover a similar exposure time and delay to that of patients with KD. The questionnaire was modified for control subjects. Instead of a date of symptom onset, subjects were provided with a randomly generated number between 2–6 weeks, and asked to select a specific event in their lives that occurred within this assigned number of weeks. Subjects were not asked to disclose what this event was, but were asked to use the event as a placeholder for “symptoms onset” to answer time-related questions. Once completed, all questionnaires were entered in a REDCap electronic capture tool hosted at The Hospital for Sick Children [20]. Upon receipt, an investigator (SO) reviewed all questionnaires to identify missing or incorrect information, and worked with the subjects to make corrections if needed and ensure that control subjects had properly understood the scenario necessary to establish the timing of “symptoms onset”.

**Table 1:** Questionnaire elements for case-control study.

Category	Specific elements
Demographics and medical history	- Ethnicity - Place of birth - Birth history (weight, gestational age) - Hospitalizations, medical events, chronic conditions - Vaccination history - Recent travel history
Family composition and medical history	- Place of birth (biological parents/grandparents) - 1^st^/2^nd^ degree relatives medical history - Recent travel history
Health up to 1 month prior to symptoms onset* and between onset and diagnosis	- Clinical signs and symptoms - Medication use - Infectious disease diagnosis
Allergies and sensitivities	- Dietary and environmental allergies - Drug/chemical sensitivity - Asthma, eczema, hay fever history
Daily exposures	- Daily activities and location - Contact with other children - Food intake (type/quantities) - Contact with pets - Neighborhood characteristics
Housing	- Number of occupants - Type and characteristics of dwelling - Chemical exposures in dwelling - Recent renovations/major modifications

* With separate sets of questions for 1–7 days prior to onset and 8–31 days prior to onset

Subjects were asked to provide their postal code and a geolocation software (Google Earth, Googleplex, Mountain View, California, USA) was used to map the immediate environment (~1 km) around the subject’s residence. Aerial pictures were taken and the distance to the specific landmarks (lakes/rivers, parks/wooded area and highways), traffic density at the closest major intersections and tree density in a 500m and 1000m radius around the residence were collected. Tree density was graded qualitatively on a scale from 1 to 5 (1 = low density, 5 = high density) independently and in a blinded manner by 3 separate investigators (SO, ML, BM) and the average of the 3 ratings was used.

The study was approved by the Research Ethics Board at the Hospital for Sick Children. All subjects provided written consent for participation in the study; a separate consent was obtained specifically for the geolocation aspect of the study. Given the depth of the questionnaire and the sensitive nature of some of the questions, subjects were allowed to decline to answer any question they did not wish to answer by crossing out the relevant questions; although no subjects availed themselves of this option.

Data from this part of the study are described as means with standard deviations, median with 25^th^ and 75^th^ percentiles and frequencies as appropriate. Comparisons between patients and controls were performed using Fisher’s exact test and Student’s t-test assuming unequal variance between samples. All statistical analyses were performed using SAS v9.4 (SAS Institute, Cary NC).

### Canadian epidemiology study

Data from all pediatric (0–18 years old) hospital admissions with a primary or secondary diagnosis of KD (ICD-10-CA standard: M30.3—Mucocutaneous lymph node syndrome [Kawasaki disease]) from March 2004 to March 2012 were obtained for Canada from the Discharge Abstract Database (DAD) maintained by the Canadian Institute for Health Information (CIHI) (except Quebec which does not report data to CIHI). Multiple admissions for a given patient were tracked by the universal provincial health number (unique to each patient), which was provided in an encrypted format. Given that transfers of patients between hospitals during the acute phase of the disease are common, and that patients with KD complicated by coronary artery aneurysms often have multiple admissions, a previously validated algorithm was used to specifically identify acute admissions and exclude all readmissions and transfers between hospitals [21]. The date of hospital admission was used as a surrogate for the date of diagnosis.

### Biological and atmospheric data

Atmospheric biology data consisting of daily measurements of major biological atmospheric particles was provided by the Aerobiology Research Laboratories (Ottawa, Canada). Particle counts from February-October, 2011, for the Greater Toronto Area (GTA) were obtained for analysis.

### Cross correlations

To compare the number of new KD cases with atmospheric particle counts, the two time series were both smoothed with a moving average with a 5-day window. A cross-correlation was then performed to evaluate the strength and direction of the time-lagged relationship between KD cases and atmospheric particle counts with lags between 0 to 25 days.

### Wind composite analysis

Monthly means of daily means of 10 meter zonal (*u*) and meridional (*v*) surface wind components were obtained from the European Centre for Medium Range Weather Forecasts (ECMWF) ERA-Interim reanalysis dataset [22]. A composite analysis was performed to determine tropospheric wind conditions associated with occurrences of KD. New KD cases were averaged over Canada, aggregated per month, smoothed with a 3-month-running mean filter and standardized. The number of new KD cases was considered high if it was larger and low if it was lower than one standard deviation above or below the multi-year monthly average number of cases, respectively. The resulting composite consists of 15 months of wind patterns for a high and 14 months for a low number of KD cases for that month. Through the accumulation and averaging of wind fields over several months with anomalous high and low KD cases in a given month, background variability in the climate system (noise) could be reduced and the signal made more easily identifiable.

#### Association between winds directionality and biological atmospheric particle measurements

Wind data were averaged over longitude 41.25°N to 45.75°N and latitude 279°E to 284.25°E to obtain daily values around the GTA. The wind component was then standardized, but not centered to keep the original definition for the direction (positive westward or westerly, negative eastward or easterly). Biological atmospheric particle measurements for the GTA were compared to wind directions over the GTA. Atmospheric particle measurements were averaged over days with westerly (n = 252) and days with easterly (n = 113) winds over Toronto.

#### Model for global distribution of KD

We proposed a framework for the individual-level risk of developing KD that would also help explain the global distribution of KD. We used incidence estimates from as many countries as possible from published reviews [2–4] and from the abstracts presented at the last two International Kawasaki Disease Symposium (Kyoto, Japan, February 7–10, 2012 and Honolulu, United-States, February 3–6, 2015). Demographics (population, urbanization, gross domestic product, ethnic composition) were obtained from the United Nations Department of Economic and Social Affairs, and geographic information (annual precipitations, latitude, longitude) from the National Oceanic and Atmospheric Administration (NOAA) and made available through Weatherbase [22]. Linear regression with backward selection of variables was used to create a model for the global distribution of KD. Incidence of KD was transformed using squared root to account for the heavily skewed distribution of KD incidence as a result of the very high incidences in Japan and Korea. Square root transformation was chosen over other modelling methods because it maintained the unique characteristics of Japan and Korea but prevented model overfitting.

## Results

### Environmental epidemiology: Patient characteristics and potential genetic risk factors (SickKids cohort)

There were 297 newly diagnosed KD patients during the study period. A total of 168 subjects were enrolled in this study (81 patients with KD (27% participation rate), 87 controls). Patients and controls were similar in terms of age (median 4.1 (2.1–6.3) years for KD vs. 3.3 (1.6–7.1) years for controls, p = 0.64) and gender (45 males (56%) for KD vs. 39 (45%) for controls, p = 0.16). As shown in Table 2, patients with KD were more likely to be of Asian origin and generally less likely to be of European ancestry. Patients with KD were found to have an older gestational age at birth (39.2±2.1 vs. 38.5±2.5 weeks, p = 0.04) and were more likely to have gestation ≥40 weeks (45/77 (58%) vs. 31/80 (39%), p = 0.02). Patients with KD were not at increased odds of reporting food/environmental sensitivity or allergy (24/81 (30%) vs. 26/86 (30%), p = 1.00), asthma (4/81 (5%) vs. 6/86 (7%), p = 0.75) or eczema (21/81 (26%) vs. 15/86 (17%), p = 0.19). There were no differences between groups regarding family history of autoimmune disorders (15/81 (19%) vs. 22/87 (25%), p = 0.35) or asthma (29/81 (36%) vs. 28/86 (32%), p = 0.63).

**Table 2:** Distribution of ethnicity/origin across controls and patients with Kawasaki disease (KD).

Ethnicity/origin*	Control (N = 87)	KD (N = 81)	p
European/European ancestry	72 (83%)	48 (59%)	0.001
Chinese, Taiwanese, Japanese, Korean	7 (8%)	25 (31%)	<0.001
African, African American, Latin American, Hispanic, Caribbean	4 (5%)	13 (16%)	0.02
Arab, Middle Eastern, North African, Indian, South Asian, Southeast Asian, Filipino	15 (17%)	9 (11%)	0.28
Other	4 (5%)	2 (3%)	0.68

* Categories are not mutually exclusive; responders were allowed to select as many categories as applied

### Environmental epidemiology: Potential modulatory factors (SickKids cohort)

Patients with KD were living in larger households than controls (4.4±1.2 vs. 3.8±0.9 occupants, p = 0.001). Patients with KD and controls were found to be nearly identical regarding contact with other children, daily activities and food. Patients with KD were generally less exposed to environmental allergens in many aspects of their daily lives (Table 3). They were more likely to primarily drink filtered or bottle water, were exposed less to household pets, were more likely to live in a dwelling constructed in the last 10 years and were less likely to live in an area with dense tree coverage, near a park, body of water or a farm. There was some statistically significant associations between the different exposures reported in Table 3. When considering all factors in the same multivariable regression model (except for tree coverage at 500 and 1000m, which were collinear to self-reported tree coverage), all exposures other than living close to a body of water remained close to or statistically significant indicating independence from each other. Patients with KD were more likely to live in dwellings that had undergone deep carpet cleaning in the month prior to KD symptoms onset (6/81 (7%) vs. 0/83 (0%), p = 0.01). There was no evidence of association between any of the chemical exposures that were collected and the odds of KD. There were some associations between exposures to environmental allergens and patients ethnicity/origins; the associations reported here remained substantially unchanged when accounting for differences in ethnicity/origins.

**Table 3:** Habitual environmental exposures in children with Kawasaki disease (KD).

	N	Control	N	KD	p
Filtered/bottled water (versus tap/well)	87	39 (45%)	79	50 (63%)	0.02
Number of pets in household (excluding fish)	87		81		0.03
None		51 (58%)		57 (70%)	
1		20 (23%)		20 (25%)	
2		16 (18%)		4 (5%)	
Dwelling age <10 years	86	18 (21%)	80	37 (46%)	0.001
Tree coverage (self-reported)	87		80		0.004
Dense		3 (4%)		7 (9%)	
Moderate		67 (77%)		42 (53%)	
Sparse		17 (20%)		31 (39%)	
Tree coverage measured at 500m*	77	2.2±0.7	66	1.9±0.8	0.009
Tree coverage measured at 1000m*	77	2.5±0.7	66	2.3±0.7	0.02
House near a body of water (self-reported)	87	38 (44%)	81	23 (28%)	0.05
House near a park (self-reported)	87	75 (86%)	81	56 (69%)	0.009
House near a farm (self-reported)	87	16 (18%)	81	6 (7%)	0.04

* 5 point Likert scale, average of three blinded assessors, see Methods for full detail

### Environmental epidemiology: Infectious disease history, exposure and vaccination (SickKids cohort)

**Fig 1:**
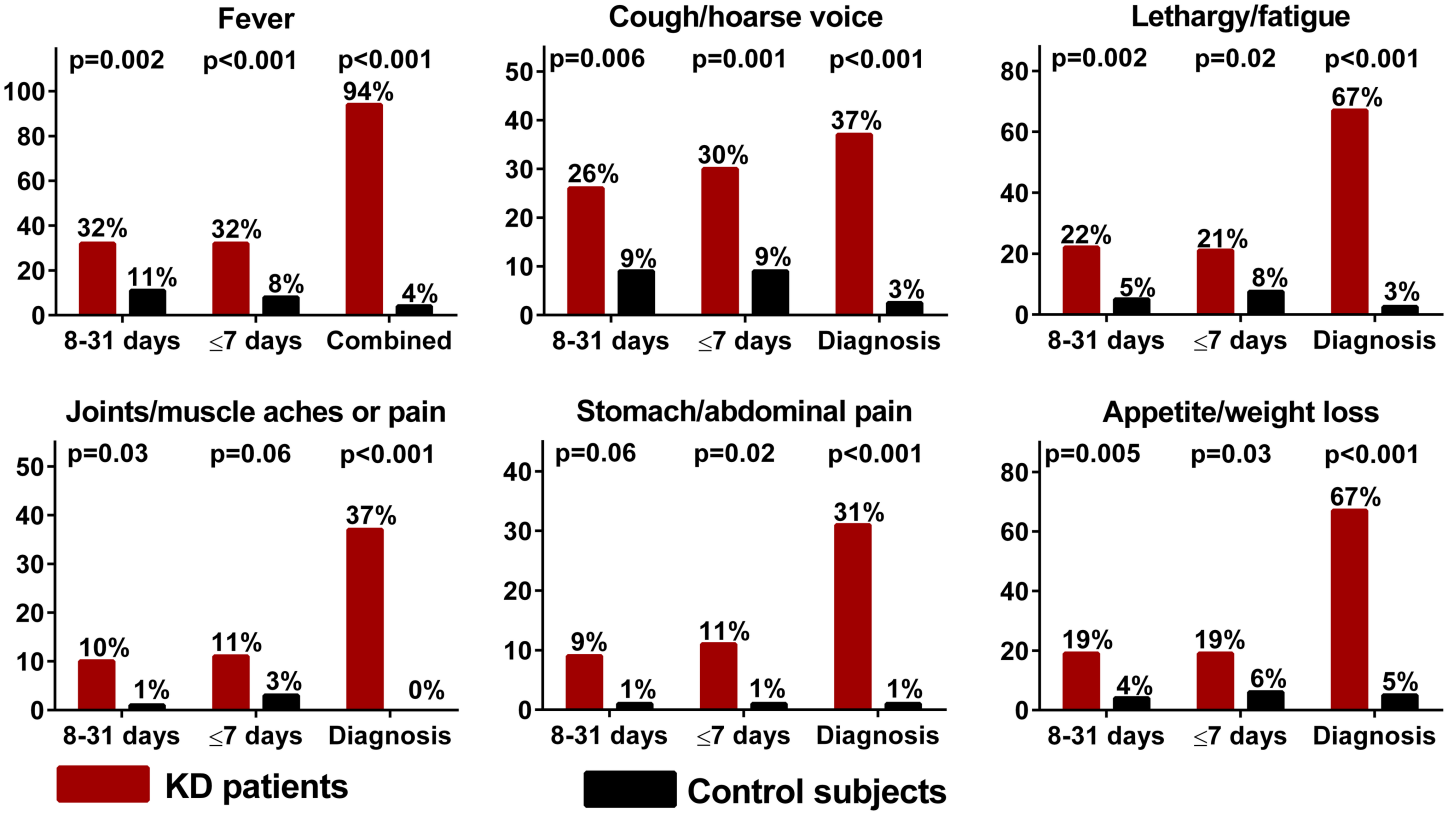
Clinical signs and symptoms in the weeks preceding the onset of Kawasaki disease (KD).

Compared to controls (data available on 80/87 (92%) controls and for all patients with KD), patients with KD were more likely to report being ill and/or affected by various and non-specific clinical signs and symptoms up to 31 days prior to their diagnosis of KD (Fig 1). However, patients with KD were not found to be at higher odds of reporting diarrhea, vomiting, earaches/otitis, jaundice, irritability or rash in this period. They were more likely to report any illness and/or feeling unwell than controls in the month prior to diagnosis of KD (1–7 days: 65% in KD vs. 29% in controls, p<0.001, 8–31 days: 64% in KD vs. 33% in controls, p<0.001). Patients with KD were more likely to report an infection confirmed by a medical practitioner in the month prior to KD symptoms onset (33% vs. 15%, p = 0.01). No pattern could be identified regarding the type of infection or timing of the diagnosis (19%, 11%, 26%, 7% at 4 weeks, 3 weeks, 2 weeks and 1 week before onset of KD symptoms respectively and the remaining 37% diagnosed after the onset of KD symptoms). Patients with KD were not found to be at increased odds of having received a recent vaccination (i.e. within 1 month) (11/78 (14%) vs. 13/77 (17%), p = 0.66). There were no differences in vaccination status between patients with KD and controls for all vaccines with the exception of rotavirus which was less frequent in children with KD (25/81 (31%) vs. 44/86 (51%), p = 0.01). Finally, patients with KD were more likely to report at least one household member being ill in the month prior to symptoms onset (25/80 (31%) vs. 12/86 (14%), p = 0.009).

### Exposures of potential modulatory and triggering agents (Greater Toronto Area data)

**Fig 2:**
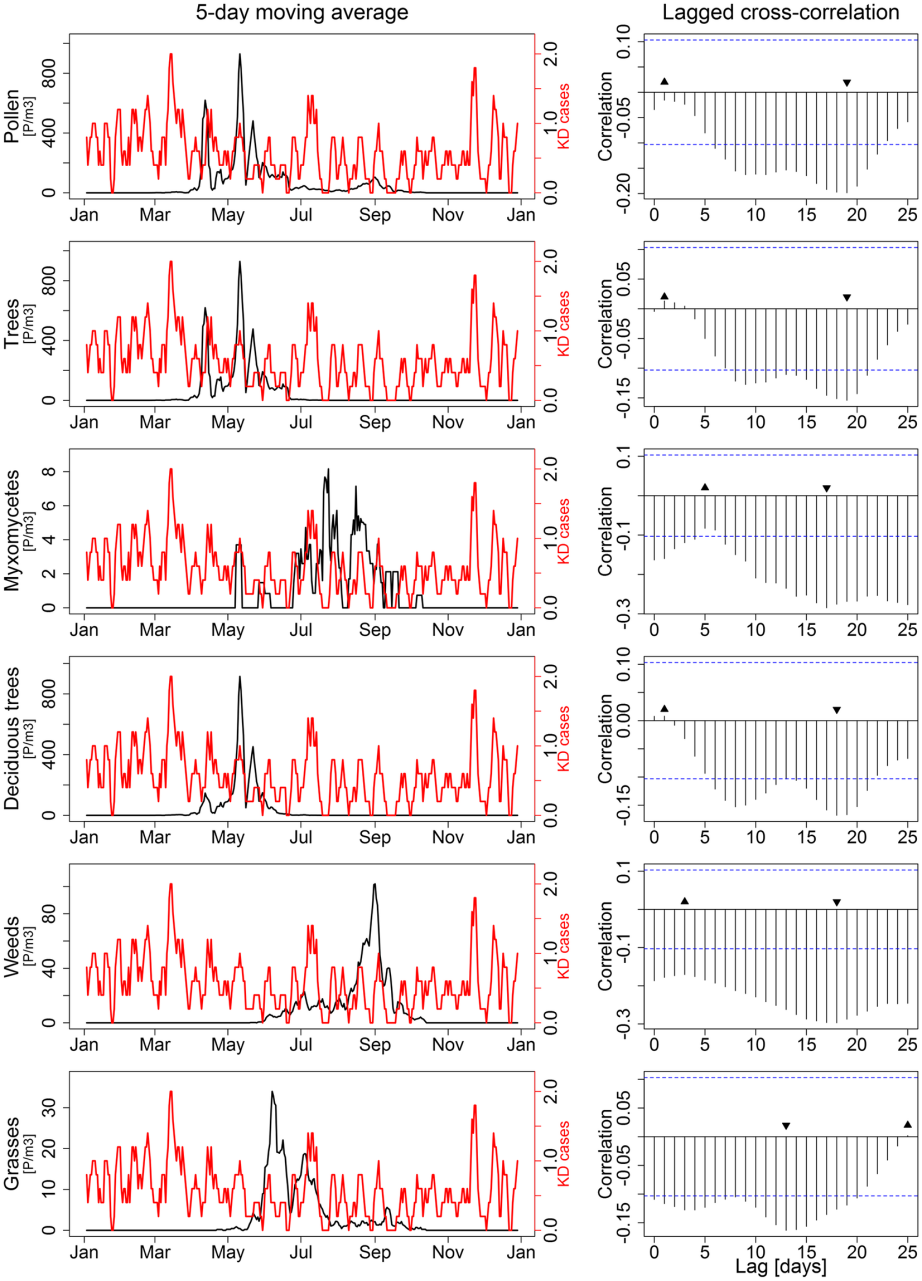
Time series (5-day moving averages) and cross-correlation of new cases of Kawasaki disease (KD) vs. count of biological atmospheric particles for lag-times of 0 to 25 days for the following types of particles: Pollen, trees (all trees and deciduous trees only reported separately), grasses, weeds and myxomycetes. Upward triangles indicate maximum and downward triangles minimum cross-correlations. The difference between lowest and peak correlation was statistically significant for all types other than grasses.

**Fig 3:**
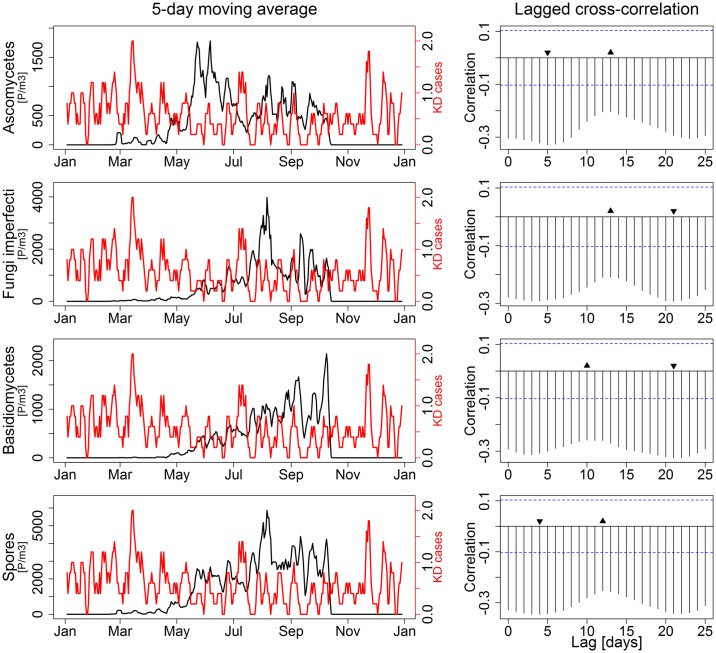
Time series (5-day moving averages) and cross-correlation of new cases of Kawasaki disease (KD) vs. count of biological atmospheric particles for lag-times of 0 to 25 days for spores and fungi (ascomycetes, basidiomycetes and fungi imperfecti separately). Upward triangles indicate maximum and downward triangles minimum cross-correlations. The difference between lowest and peak correlation was statistically significant only for ascomycetes but the overall patterns was conserved for all types.

We analyzed the number of KD cases from the CIHI database and total biological atmospheric particle counts over the GTA for the year 2011. The total number of cases in the GTA during that year totalled 208, and ranged between zero and three cases per day. We calculated the cross-correlation (the degree to which 2 time series correlate with each other) between biological atmospheric particle counts and number of cases of KD for all possible lag-times between 0 and 25 days (the maximum lag time proposed for KD). Atmospheric particles from pollen, trees (all trees and deciduous tree separately), weeds, grasses and myxomycetes showed a similar qualitative pattern, and a maximum negative cross correlation at lag-time of 14 to 21 days (Fig 2). The variation (difference minimum to peak correlation) in cross-correlation during the 0 to 25 day period was statistically significant for all categories other than grasses. Conversely, particles from fungi (ascomycetes, basidiomycetes, fungi imperfecti) and spores showed the exact opposite pattern with the lowest cross correlations at lag-times of 14 to 21 days (Fig 3). In this case, only the difference between lowest and peak cross-correlation for ascomycetes was statistically significant, but the overall patterns were qualitatively similar.

**Fig 4:**
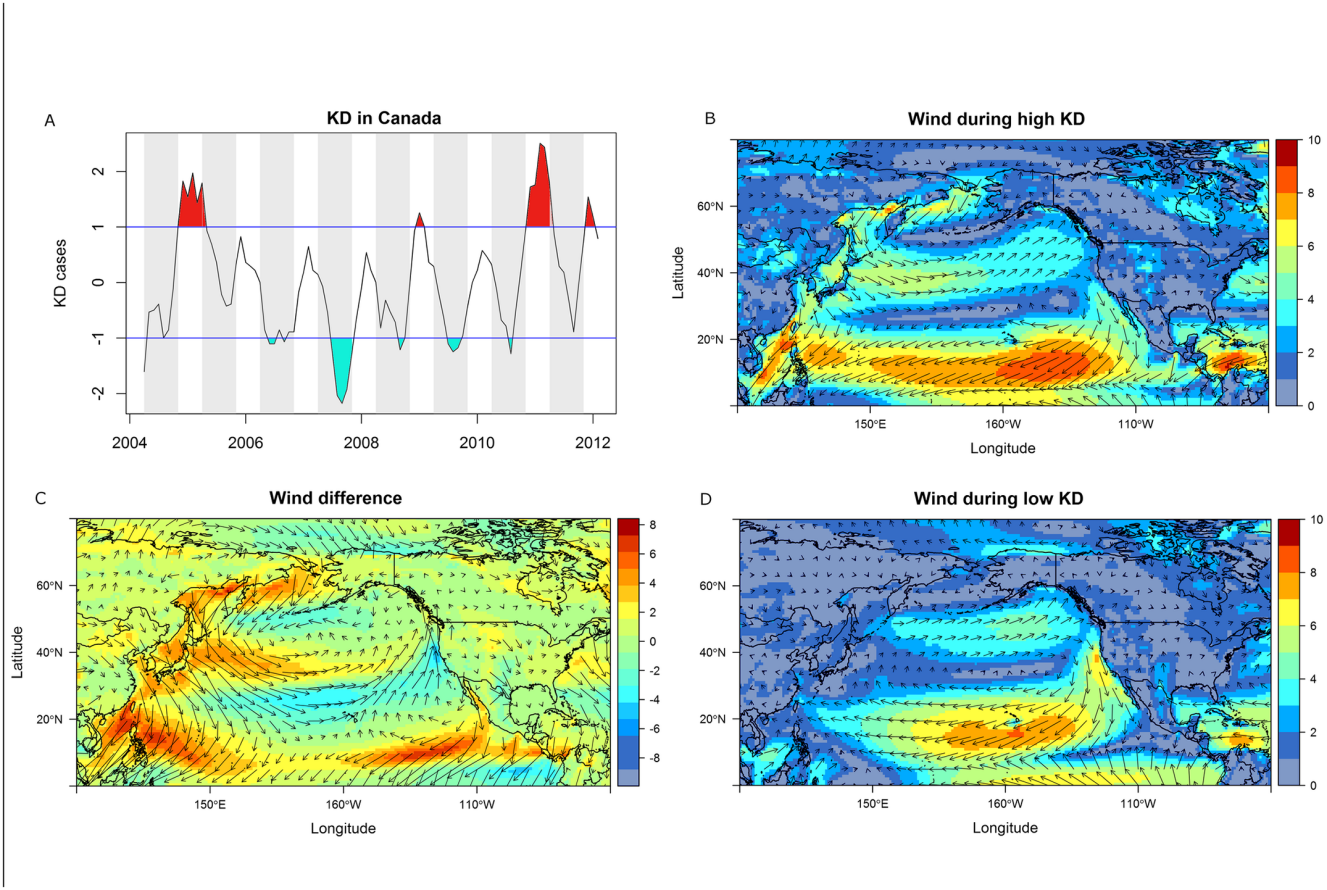
Composite analysis of wind patterns for months with high and low Kawasaki disease (KD) occurrences. Time series of standardized monthly number of KD cases, with months (May-October in grey) and anomalously high and low occurrences of new KD cases highlighted in red and blue, respectively (A). Wind speed (color, units [m/s]) and direction (arrows) during months with high KD occurrences (B), low KD occurrences (D) and difference between months with high and low KD occurrences (C) centered over the Pacific Ocean.

A time series analysis of the incidence of KD cases across Canada is shown in Fig 4A. Anomalously high numbers of new KD cases seemed to cluster during the winter months (November to April), while lower numbers were found during warmer months (May to October). This seasonal pattern was strongly and negatively associated with the level of biological atmospheric particles in Canada (Figs 2 and 3).

### Potential source for triggering agent (Canada-wide data)

In order to assess whether it might be possible that periods of high KD case numbers in Canada were related to an environmental trigger from China and Japan as suggested by Rodo *et al*. [18] for San Diego, we investigated the dynamics of KD and possible associated wind patterns over the Pacific Ocean. During months with high numbers of KD cases in Canada, westerly winds over the Pacific Ocean were stronger, hence, might have been able to transport a potential trigger for KD from Asia to North America and Canada (Fig 4B–4D). Note that these wind patterns are typical for winter and summer months, with the highest wind speeds observed during winter months in the northern mid-latitudes [23]. The winds over Canada are westerly during both high and low KD seasons (Fig 4B and 4D).

In consideration of the Rodo *et al*. hypothesis, we also explored the association between wind direction and the count of fungi imperfecti (the category that would include fungal toxins and *Candida* fungi) in the atmosphere [17]. This analysis showed that in the GTA, westerly winds were associated with an increase in the atmospheric measurement of fungi imperfecti (average of 920.3 P/m^3^ during westerlies vs. 476.7 P/m^3^ during easterlies, p = 0.002). The magnitude of the increase in fungi imperfecti associated with westerly winds was the highest of all types of atmospheric biological particles investigated in this study.

We used regression analysis to determine whether the various non-environmental and environmental factors were associated with the global distribution of KD. For each of the 41 countries with available estimates of incidence, we analyzed several elements. To model genetic predisposition we included the proportion of the population from Japanese, Korean or Chinese ancestry. The proportion of country urbanization and gross national product were used as surrogates for environmental allergens exposure (increase in either would be associated with lower exposure). Finally, western and southern distance from 100.00°E/60.00°N (Northeastern China) was calculated to simulate the global distribution and deposition of a possible trigger originating in dust from Northeastern China and transported through tropospheric wind. The results of our model are presented in Table 4; in its current form, the model was found to explain 84% of the variation in the global incidence of KD.

**Table 4:** Factors associated with global distribution of Kawasaki disease (KD) (model R^2^: 0.84, p<0.001).

	EST (95% CI)*	p-value
Proportion of population of Asian ancestry	0.626 (0.112)	<0.001
Proportion of country that is urbanized (per 10%)	0.928 (0.265)	0.002
Gross national product (per USD $10,000, 1/x transformation)	2.035 (0.712)	0.008
Interaction of urbanization and gross national product	-0.407 (0.127)	0.003
Westward degrees of longitude from 100.00°E (per 10°)	-0.843 (0.232)	0.001
Southern degrees of latitude from 60.00°N (per 10°)	-0.204 (0.050)	<0.001
Interaction of longitude and latitude distance from 100.00°E/60.00°N	0.039 (0.015)	0.01

* √ of incidence per 100,000 children 0–4 years old per year

## Discussion

**Fig 5:**
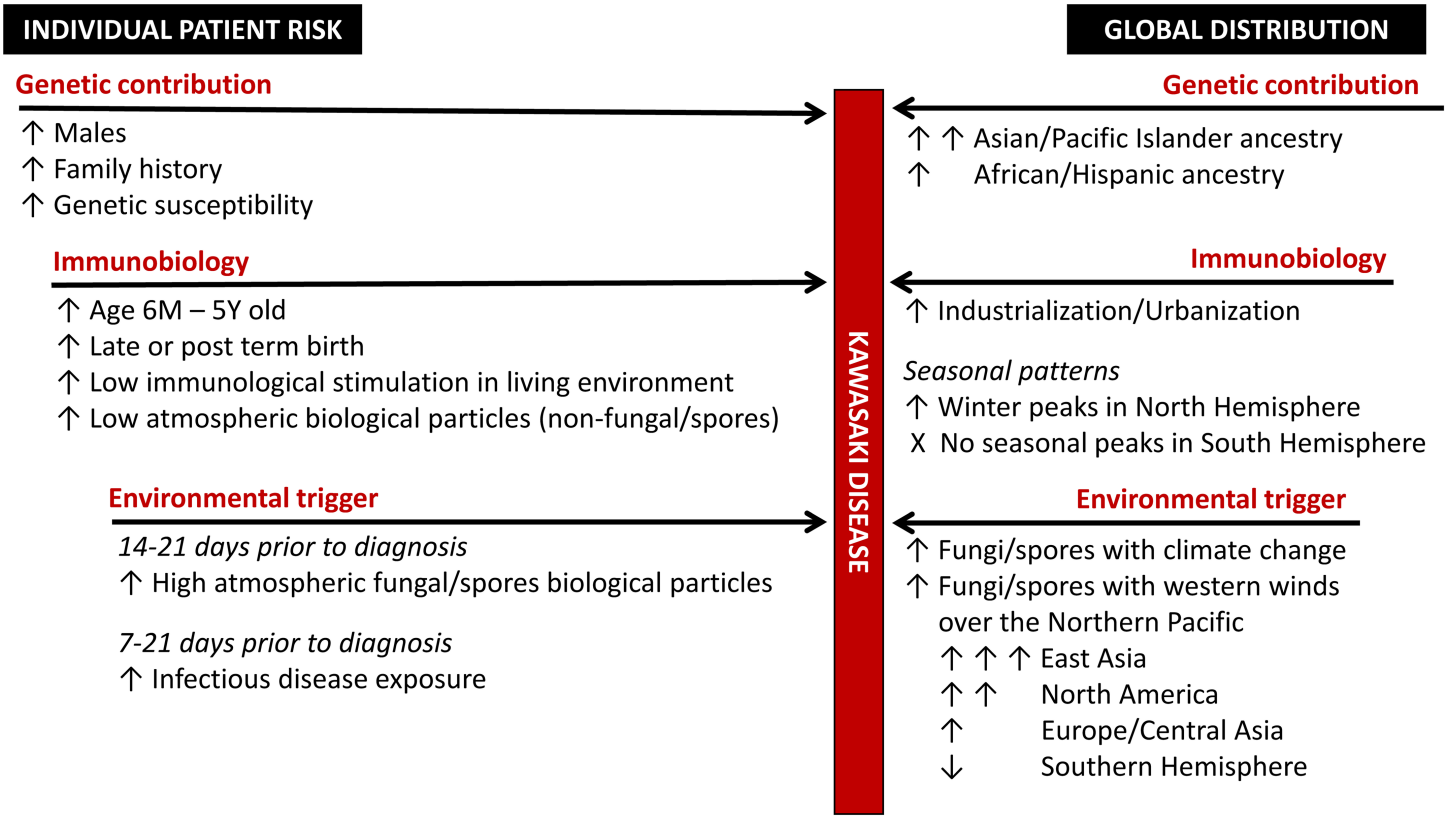
Conceptual framework for individual risk of developing Kawasaki disease (KD) linking disease etiology, pathogenesis and global distribution.

The search to explain the etiology and pathogenesis of KD continues, despite more than 40 years of investigation. Many associations between the incidence of KD and genetic, environmental or infectious factors have previously been described. However, most associations have only been shown at the local level and have failed to integrate into a framework that might explain the global epidemiology of the disease. Through the various aspects of this study, we investigated the environmental epidemiology of KD and have proposed a new framework that attempts to explain the global distribution of KD. Our proposed framework (Fig 5) includes three separate elements: 1) the proportion of the population that have the highest genetic susceptibility to KD, 2) modulation of risk, determined by habitual exposure to environmental allergens, seasonal presence/absence of atmospheric biological particles and presence of an infectious diseases process, and 3) exposure to the still unidentified disease trigger. Importantly, our data and proposed framework support the novel paradigm proposed by Rodo *et al*. [24], whereby the trigger for KD is not an infectious agent but another environmental trigger, potentially a fungal toxin. The different elements of this framework are supported by the data presented herein, but will also be contextualized in the current knowledge base available on the topic of KD.

A genetic susceptibility to KD has long been established, as evidenced by multiple genetic studies [8–11]; the significantly higher incidence in people of East Asian ancestry, and potentially of African ancestry, as seen in this study and others, regardless of their country of residence [3, 4, 25]; the presence of multi-generational familial clustering [26]; and the elevated risk of recurrence versus risk of first episode in a KD-naïve child [5–7].

In our proposed framework, we included the concept of modulation, which encompasses many of the environmental or infectious factors that have previously been hypothesized to be the potential trigger of KD. In this proposed framework, environmental factors would influence, over time, the susceptibility of children to develop KD when encountering the disease trigger, but these factors would not directly act as the trigger. The mechanism for modulation could be either an immunological process, such as maturation, an alteration in the microbiome or both [24]. Based on this study, we have identified 4 potential modulating factors: early development of the immune system, habitual exposure to environmental allergens, temporal exposure to biological atmospheric particles, and temporal experience of a stimulatory or co-stimulatory event, potentially of infectious origin.

Late or post term birth [27] and not being breastfed as an infant [28] are known to be associated with multiple immunological diseases, including allergies, which are themselves associated with an increased risk of KD [29–31]. The association between late or post term birth and increased risk of KD was also observed in the present study. Based on our results, habitual exposure to environmental allergens might play a role in the modulation of risk of KD. Patients with KD were found to live in environments generally less exposed to environmental allergens. This effect was limited to allergens of biological origin as opposed to pollution-driven chemicals; both in this study and in previous ones, these have been shown to not be associated with risk of KD [32, 33]. This pattern of decreased exposure to the biological environment in childhood and increased risk of diseases of the immune system is consistent with the *hygiene hypothesis*. This has been linked to many diseases including, asthma and allergies, the incidence of which have significantly increased since the 1960’s, as has the incidence of KD. Further supporting this theory is the observation from recent epidemiological data that the incidence of KD, outside of Japan and Korea, has been more or less stable in developed countries but that it is increasing in countries that are rapidly industrializing [3]. A recent review of the epidemiological information available regarding KD has also noted the consistency between epidemiological risk factors for KD and the *hygiene hypothesis* [34].

The third element of our modulation framework is the temporal association between exposure to biological atmospheric particles and risk of KD. In this study, we have found that exposures to pollen temporally reduced the risk of KD, regardless of the type of pollen being present in the atmosphere. This association could at least partially explain the seasonal patterns observed for KD. Using data from an international consortium, Burns, *et al*. showed winter peaks in the Northern Hemisphere and a lack of seasonality in the tropical areas and the Southern Hemisphere [35]. Hypothesizing the presence of pollen in the local atmosphere is protective against the trigger for KD, it would make sense that winter peaks are observed in the Northern Hemisphere, at a time where there is little or no pollen in the atmosphere. In contrast, tropical zones and the Southern Hemisphere have much more stable weather patterns throughout the year, and pollen from various origins is constantly present in the atmosphere.

The final element of our framework is exposure to the disease trigger. Until recently, an infectious trigger had been hypothesized to be responsible for KD due to the seasonal patterns [12, 35], non-random case clustering [36–38], the common co-occurrence of KD and infectious symptoms or infections [13, 39, 40] and the common presence of sick household members immediately preceding KD diagnosis [41]. We have found in this study an increased burden of infectious disease and the presence of sick family members prior to the diagnosis of KD, and a high proportion of patients with infectious disease symptoms. Temporal clustering of cases in geographically unrelated areas is not consistent with person-to-person area, which would be typical of an infectious process [37]. No infectious agent is known to have a global distribution pattern similar to KD [24, 42], and there is no consistency in the type of infectious disease reported to co-occur with KD. These inconsistencies have led many to conclude that multiple infectious agents could trigger KD [36, 39, 41, 42]. An alternative explanation could be that infections are modulating factors rather than the trigger or cause of the disease. This is what we propose in our global distribution framework.

Environmental epidemiology studies from the 1980-1990s have shown that multiple exposures/events 14–21 days preceding symptoms onset could potentially trigger KD [14–16, 43]. Recent studies have suggested that epidemiological patterns in Japan, Hawaii and the United-States might be associated with exposure to fungal toxins or other environmental factors originating from Northeastern China [17, 18], the distribution of which might be influenced by global climate patterns [44]. A similar observation has been found in Chile, where epidemiological data suggest that the triggering agent could be transported from the Atacama Desert through tropospheric winds [45]. Although not designed to answer specifically this question, the association between wind direction distribution and atmospheric fungus concentrations, as found in this study, is consistent with the theory. Given that some animal models of KD use *Candida* species to trigger a KD-like syndrome and that *Candida* infection is known to be associated with asthma episodes, it is conceivable that a fungal toxin could be the trigger of KD. Under this assumption, viral and bacterial infections would act as modulating agents, along with atmospheric biological particles, in the weeks before the exposure of a susceptible child to the trigger and the appearance of KD symptoms.

Taken together, these factors were combined into a global framework, where the risk of KD was determined by the four concurrent processes described above: genetic susceptibility, long and short-term modulation of risk and exposure to the disease trigger. Although the model we propose is theoretical and only uses gross estimation of the disease incidence and risk factors, it nevertheless was able to account for more than 80% of the variation in the global distribution of KD. This model should be refined further as incidence data from more countries become available.

This study must be viewed in light of some limitations. First, the majority of the data used are limited to the Canadian context, and the environmental associations identified in this study should be re-assessed in a global context. Our environmental epidemiology questionnaire might be subject to recall and self-report bias, and we cannot be certain that some important environmental risk factors have not been included. Given the sample size available for the case-control part of the study, it is possible that some comparisons between patients and controls might have been underpowered; particularly in the case of rare features. Our analysis of atmospheric biological particles should be seen as being purely exploratory, given that these data were available only for a single year and for the GTA. While we hypothesize that *Candida* fungi might have an important role in the etiology of KD, our atmospheric particle data did not individually measure it, but rather included it in a larger group of fungi and we cannot exclude potentially contributing environmental factors which were not measured; additionally, atmospheric data from the west coast of Canada; the region at highest theoretical exposure to *Candida* fungi were not available to confirm this hypothesis. The associations described are only associations and not cause and effect, with the potential mechanisms logical but only speculative. Finally, both our risk framework and global distribution models may represent an ecologic fallacy, and not the results of a single experimental model.

In conclusion, this study described the environmental epidemiology of KD, both at the individual patient level and in a large geographic area. We used these data to propose a theoretical framework, which we tested against the global distribution of the disease and found that a substantial proportion in the variance of KD incidence between countries was accounted by our framework. Future research should attempt to replicate the associations observed in this study, and the predictive model for KD incidence should be revised as additional countries report their epidemiological data.

## References

[1] McCrindle BW, Rowley AH, Newburger JW, Burns JC, Bolger AF, Gewitz M, et al Diagnosis, Treatment, and Long-Term Management of Kawasaki Disease: A Scientific Statement for Health Professionals From the American Heart Association. Circulation. 2017;135(17):e927–e99. doi: 10.1161/CIR.0000000000000484 . 28356445 10.1161/CIR.0000000000000484

[2] Nakamura Y, Yanagawa H. The worldwide epidemiology of Kawasaki disease. Progress in Pediatric Cardiology. 2004;19:99–108.

[3] Singh S, Vignesh P, Burgner D. The epidemiology of Kawasaki disease: a global update. Archives of disease in childhood. 2015;100(11):1084–8. Epub 2015/06/27. doi: 10.1136/archdischild-2014-307536 . 26111818 10.1136/archdischild-2014-307536

[4] Uehara R, Belay ED. Epidemiology of Kawasaki disease in Asia, Europe, and the United States. Journal of epidemiology. 2012;22(2):79–85. Epub 2012/02/07. doi: 10.2188/jea.JE20110131 . 22307434 10.2188/jea.JE20110131 PMC3798585

[5] Sudo D, Nakamura Y. Nationwide surveys show that the incidence of recurrent Kawasaki disease in Japan has hardly changed over the last 30 years. Acta Paediatr. 2017;106(5):796–800. doi: 10.1111/apa.13773 . 28164356 10.1111/apa.13773

[6] Maddox RA, Holman RC, Uehara R, Callinan LS, Guest JL, Schonberger LB, et al Recurrent Kawasaki disease: USA and Japan. Pediatr Int. 2015;57(6):1116–20. doi: 10.1111/ped.12733 . 26096590 10.1111/ped.12733 PMC4676732

[7] Chahal N, Somji Z, Manlhiot C, Clarizia NA, Ashley J, Yeung RS, et al Rate, associated factors and outcomes of recurrence of Kawasaki disease in Ontario, Canada. Pediatr Int. 2012;54(3):383–7. doi: 10.1111/j.1442-200X.2012.03628.x . 22631567 10.1111/j.1442-200X.2012.03628.x

[8] Khor CC, Davila S, Shimizu C, Sheng S, Matsubara T, Suzuki Y, et al Genome-wide linkage and association mapping identify susceptibility alleles in ABCC4 for Kawasaki disease. J Med Genet. 2011;48(7):467–72. doi: 10.1136/jmg.2010.086611 . 21571869 10.1136/jmg.2010.086611

[9] Onouchi Y, Gunji T, Burns JC, Shimizu C, Newburger JW, Yashiro M, et al ITPKC functional polymorphism associated with Kawasaki disease susceptibility and formation of coronary artery aneurysms. Nat Genet. 2008;40(1):35–42. Epub 2007/12/18. doi: 10.1038/ng.2007.59 . 18084290 10.1038/ng.2007.59 PMC2876982

[10] Onouchi Y, Ozaki K, Buns JC, Shimizu C, Hamada H, Honda T, et al Common variants in CASP3 confer susceptibility to Kawasaki disease. Hum Mol Genet. 2010;19(14):2898–906. doi: 10.1093/hmg/ddq176 . 20423928 10.1093/hmg/ddq176 PMC2893807

[11] Onouchi Y, Ozaki K, Burns JC, Shimizu C, Terai M, Hamada H, et al A genome-wide association study identifies three new risk loci for Kawasaki disease. Nat Genet. 2012;44(5):517–21. doi: 10.1038/ng.2220 . 22446962 10.1038/ng.2220

[12] Nakamura Y, Yashiro M, Uehara R, Oki I, Watanabe M, Yanagawa H. Monthly observation of the number of patients with Kawasaki disease and its incidence rates in Japan: chronological and geographical observation from nationwide surveys. J Epidemiol. 2008;18(6):273–9. Epub 2008/12/17. doi: 10.2188/jea.JE2008030 . 19075496 10.2188/jea.JE2008030 PMC4771612

[13] Benseler SM, McCrindle BW, Silverman ED, Tyrrell PN, Wong J, Yeung RS. Infections and Kawasaki disease: implications for coronary artery outcome. Pediatrics. 2005;116(6):e760–6. Epub 2005/12/03. doi: 10.1542/peds.2005-0559 . 16322132 10.1542/peds.2005-0559

[14] Fatica NS, Ichida F, Engle MA, Lesser ML. Rug shampoo and Kawasaki disease. Pediatrics. 1989;84(2):231–4. Epub 1989/08/01. . 2748249

[15] Daniels SR, Specker B. Association of rug shampooing and Kawasaki disease. J Pediatr. 1991;118(3):485–8. Epub 1991/03/01. . 1999796 10.1016/s0022-3476(05)82173-0

[16] Rauch AM, Glode MP, Wiggins JW Jr., Rodriguez JG, Hopkins RS, Hurwitz ES, et al Outbreak of Kawasaki syndrome in Denver, Colorado: association with rug and carpet cleaning. Pediatrics. 1991;87(5):663–9. Epub 1991/05/01. . 2020511

[17] Rodo X, Curcoll R, Robinson M, Ballester J, Burns JC, Cayan DR, et al Tropospheric winds from northeastern China carry the etiologic agent of Kawasaki disease from its source to Japan. Proc Natl Acad Sci U S A. 2014;111(22):7952–7. doi: 10.1073/pnas.1400380111 . 24843117 10.1073/pnas.1400380111 PMC4050536

[18] Rodo X, Ballester J, Cayan D, Melish ME, Nakamura Y, Uehara R, et al Association of Kawasaki disease with tropospheric wind patterns. Sci Rep. 2011;1:152 doi: 10.1038/srep00152 . 22355668 10.1038/srep00152 PMC3240972

[19] Carsley S, Borkhoff CM, Maguire JL, Birken CS, Khovratovich M, McCrindle B, et al Cohort Profile: The Applied Research Group for Kids (TARGet Kids!). Int J Epidemiol. 2015;44(3):776–88. doi: 10.1093/ije/dyu123 . 24982016 10.1093/ije/dyu123 PMC4521122

[20] Harris PA, Taylor R, Thielke R, Payne J, Gonzalez N, Conde JG. Research electronic data capture (REDCap)—a metadata-driven methodology and workflow process for providing translational research informatics support. Journal of biomedical informatics. 2009;42(2):377–81. Epub 2008/10/22. doi: 10.1016/j.jbi.2008.08.010 . 18929686 10.1016/j.jbi.2008.08.010 PMC2700030

[21] Manlhiot C, O’Shea S, Bernknopf B, Labelle M, Chahal N, Dillenburg RF, et al Epidemiology of Kawasaki Disease in Canada 2004–2014: Validation of passive surveillance through administrative data. CMAJ. 2017:(in press). 10.1016/j.cjca.2017.12.009 29395706

[22] Dee DP U S, Simmons AJ, Berrisford P, Poli P, Kobayashi S, et al The ERA-Interim reanalysis: Configuration and performance of the data assimilation system. Quarterly Journal of the Royal Meteorological Society. 2011;137(656):553–97.

[23] Sandwell DT, Agreen RW. Seasonal variation in wind speed and sea state from global satellite measurements. Journal of Geophysical Research: Oceans. 1984;89(C2):2041–51.

[24] Rodo X, Ballester J, Curcoll R, Boyard-Micheau J, Borras S, Morgui JA. Revisiting the role of environmental and climate factors on the epidemiology of Kawasaki disease. Ann N Y Acad Sci. 2016;1382(1):84–98. doi: 10.1111/nyas.13201 . 27603178 10.1111/nyas.13201

[25] Okubo Y, Nochioka K, Sakakibara H, Testa M, Sundel RP. National survey of pediatric hospitalizations due to Kawasaki disease and coronary artery aneurysms in the USA. Clin Rheumatol. 2017;36(2):413–9. doi: 10.1007/s10067-016-3512-6 . 27987089 10.1007/s10067-016-3512-6

[26] Fujita Y, Nakamura Y, Sakata K, Hara N, Kobayashi M, Nagai M, et al Kawasaki disease in families. Pediatrics. 1989;84(4):666–9. Epub 1989/10/01. . 2780128

[27] Crump C, Sundquist K, Sundquist J, Winkleby MA. Gestational age at birth and risk of allergic rhinitis in young adulthood. J Allergy Clin Immunol. 2011;127(5):1173–9. doi: 10.1016/j.jaci.2011.02.023 . 21439628 10.1016/j.jaci.2011.02.023 PMC3085668

[28] Yorifuji T, Tsukahara H, Doi H. Breastfeeding and Risk of Kawasaki Disease: A Nationwide Longitudinal Survey in Japan. Pediatrics. 2016;137(6). doi: 10.1542/peds.2015-3919 . 27244853 10.1542/peds.2015-3919

[29] Hwang CY, Hwang YY, Chen YJ, Chen CC, Lin MW, Chen TJ, et al Atopic diathesis in patients with Kawasaki disease. J Pediatr. 2013;163(3):811–5. doi: 10.1016/j.jpeds.2013.03.068 . 23647775 10.1016/j.jpeds.2013.03.068

[30] Tsai YJ, Lin CH, Fu LS, Fu YC, Lin MC, Jan SL. The association between Kawasaki disease and allergic diseases, from infancy to school age. Allergy Asthma Proc. 2013;34(5):467–72. doi: 10.2500/aap.2013.34.3697 . 23998245 10.2500/aap.2013.34.3697

[31] Wei CC, Lin CL, Kao CH, Liao YH, Shen TC, Tsai JD, et al Increased risk of Kawasaki disease in children with common allergic diseases. Ann Epidemiol. 2014;24(5):340–3. doi: 10.1016/j.annepidem.2014.02.003 . 24613197 10.1016/j.annepidem.2014.02.003

[32] Jung CR, Chen WT, Lin YT, Hwang BF. Ambient Air Pollutant Exposures and Hospitalization for Kawasaki Disease in Taiwan: A Case-Crossover Study (2000–2010). Environ Health Perspect. 2017;125(4):670–6. doi: 10.1289/EHP137 . 27458717 10.1289/EHP137 PMC5381970

[33] Zeft AS, Burns JC, Yeung RS, McCrindle BW, Newburger JW, Dominguez SR, et al Kawasaki Disease and Exposure to Fine Particulate Air Pollution. J Pediatr. 2016;177:179–83 e1. doi: 10.1016/j.jpeds.2016.06.061 . 27496266 10.1016/j.jpeds.2016.06.061

[34] Rigante D, Tarantino G, Valentini P. Non-infectious makers of Kawasaki syndrome: tangible or elusive triggers? Immunol Res. 2016;64(1):51–4. doi: 10.1007/s12026-015-8679-4 . 26232895 10.1007/s12026-015-8679-4

[35] Burns JC, Herzog L, Fabri O, Tremoulet AH, Rodo X, Uehara R, et al Seasonality of Kawasaki disease: a global perspective. PLoS One. 2013;8(9):e74529 doi: 10.1371/journal.pone.0074529 . 24058585 10.1371/journal.pone.0074529 PMC3776809

[36] Kao AS, Getis A, Brodine S, Burns JC. Spatial and temporal clustering of Kawasaki syndrome cases. Pediatr Infect Dis J. 2008;27(11):981–5. doi: 10.1097/INF.0b013e31817acf4f . 18852687 10.1097/INF.0b013e31817acf4f PMC2870532

[37] Burns JC, Cayan DR, Tong G, Bainto EV, Turner CL, Shike H, et al Seasonality and temporal clustering of Kawasaki syndrome. Epidemiology. 2005;16(2):220–5. . 15703537 10.1097/01.ede.0000152901.06689.d4 PMC2894624

[38] Sano T, Makino N, Aoyama Y, Ae R, Kojo T, Kotani K, et al Temporal and geographical clustering of Kawasaki disease in Japan: 2007–2012. Pediatr Int. 2016;58(11):1140–5. doi: 10.1111/ped.12970 . 26940079 10.1111/ped.12970

[39] Chang LY, Lu CY, Shao PL, Lee PI, Lin MT, Fan TY, et al Viral infections associated with Kawasaki disease. J Formos Med Assoc. 2014;113(3):148–54. doi: 10.1016/j.jfma.2013.12.008 . 24495555 10.1016/j.jfma.2013.12.008 PMC7125523

[40] Turnier JL, Anderson MS, Heizer HR, Jone PN, Glode MP, Dominguez SR. Concurrent Respiratory Viruses and Kawasaki Disease. Pediatrics. 2015;136(3):e609–14. doi: 10.1542/peds.2015-0950 . 26304824 10.1542/peds.2015-0950

[41] Tsai HC, Chang LY, Lu CY, Shao PL, Fan TY, Cheng AL, et al Transmission of acute infectious illness among cases of Kawasaki disease and their household members. J Formos Med Assoc. 2015;114(1):72–6. doi: 10.1016/j.jfma.2014.07.005 . 25205598 10.1016/j.jfma.2014.07.005

[42] Pitzer VE, Burgner D, Viboud C, Simonsen L, Andreasen V, Steiner CA, et al Modelling seasonal variations in the age and incidence of Kawasaki disease to explore possible infectious aetiologies. Proc Biol Sci. 2012;279(1739):2736–43. doi: 10.1098/rspb.2011.2464 . 22398170 10.1098/rspb.2011.2464 PMC3367771

[43] Wong D, Nutting A, Yeung RS, McCrindle BW. Kawasaki disease and scald injuries: a possible association. Can J Cardiol. 2004;20(11):1147–9. . 15457311

[44] Ballester J B J, Cayan D, Nakamura Y, Uehara R, Rodó X. Kawasaki disease and ENSO-driven wind circulation. Geophysical Research Letters. 2013;40(10):2284–9.

[45] Jorquera H, Borzutzky A, Hoyos-Bachiloglu R, Garcia A. Association of Kawasaki disease with tropospheric winds in Central Chile: is wind-borne desert dust a risk factor? Environ Int. 2015;78:32–8. doi: 10.1016/j.envint.2015.02.007 . 25743034 10.1016/j.envint.2015.02.007

